# Optimization and Comparison of Five Methods for Extraction of Coniferyl Ferulate from *Angelica sinensis*

**DOI:** 10.3390/molecules14010555

**Published:** 2009-01-23

**Authors:** Jing-Jing Xie, Jia Lu, Zheng-Ming Qian, Yue Yu, Jin-Ao Duan, Shao-Ping Li

**Affiliations:** 1 Institute of Chinese Medical Sciences, University of Macau, Macao, P.R. China; 2 Jiangsu Key Laboratory for TCM Formulae Research, Nanjing University of Chinese Medicine, Nanjing, Jiangsu 210029, P.R. China

**Keywords:** Hydrodistillation, Supercritical fluid extraction, Pressurized liquid extraction, *Angelica sinensis*, Coniferyl ferulate, Sonication.

## Abstract

Coniferyl ferulate, which is noted for its multiple pharmacological activities and chemical instability, is abundant in *Angelica sinensis*. In this paper, five methods, namely sonication extraction (SE), pressurized liquid extraction (PLE), supercritical fluid extraction (SFE), hydrodistillation (HD) and decoction (DC) for extraction of coniferyl ferulate, as well as ferulic acid, *Z/E*-ligustilide and *Z*/*E*-butylidenephthalide, from *A. sinensis* were optimized and compared. The results showed that the order of extraction efficiency was: PLE≈SE>SFE>>HD, DC. The compositions of the SE, PLE and SFE extracts, which had a high ratio of coniferyl ferulate, were very similar, while no coniferyl ferulate was obtained by HD and DC, though they had high selectivity for the extraction of ligustilide and ferulic acid, respectively. It was noteworthy that the content of ligustilide and coniferyl ferulate was not detectable in the decoction, the commonly used oral administration form of Traditional Chinese Medicines in clinical practice.

## Introduction

**Figure 1:**
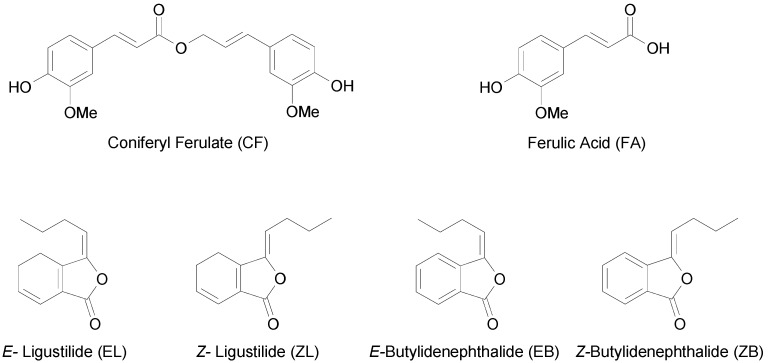
The structures of six investigated compounds in *Angelica sinensis.*

*Angelica sinensis*, known as Danggui in China, has been one of the most commonly used Traditional Chinese Medicines (TCM) for about 2,000 years. It is recommended in clinical practice as a kind of tonic, hemopoetic, spasmolytic, and analgesic drug, which is applied to tonify the blood and to treat irregular menstruation, amenorrhea, dysmenorrheal, anemia, constipation, cardiovascular disease and hepatic fibrosis [1,2,3]. In addition, modern pharmacological studies have also shown that *A. sinensis* has anti-inflammatory, antioxidant, immuno-stimulative, anti-platelet aggregation and anticancer activities [4,5,6,7,8,9]. Until now, over 70 compounds have been isolated and identified from *A. sinensis* [10]. Ferulic acid, ligustilide and other phthalides such as butylidenephthalide are usually considered as biologically active components [11,12,13,14]. Ferulic acid in particular is usually used as one of the marker compounds to assess the quality of *A. sinensis* [3], but ferulic acid is rarely found as the free form in plants [15]. Actually, its ester, coniferyl ferulate, which is abundant in *A. sinensis* [16], also has multiple pharmacological activities such as anticancer [17], antibacterial [18], vasodilating [19] and antioxidant effects [20]. Unfortunately, coniferyl ferulate, is unstable during extraction and is readily hydrolyzed into ferulic acid [16]. The extraction of coniferyl ferulate from *A. sinensis* has not been optimized though the solvent selection was investigated [16,21]. This study mainly focuses on the optimized extraction of coniferyl ferulate from *A. sinensis* using five different methods, including hydrodistillation (HD), sonication (SE), decoction (DC), supercritical fluid extraction (SFE) and pressurized liquid extraction (PLE). Besides, the content of ferulic acid, *E/Z*-ligustilide and *E/Z*-butylidenephthalide (Figure 1) in the five different extracts were also compared.

## Results and Discussion

### Optimization of sonication extraction

The parameters, including the type (acetonitrile or methanol-formic acid=95:5) and volume (30, 40, 50 or 60-fold sample weight) of solvent, as well as extraction time (30, 40 or 50 min), were investigated to optimize SE of coniferyl ferulate from *A. sinensis* using a univariate approach. The extraction temperature was controlled at room temperature because coniferyl ferulate is heat unstable. Methanol-formic acid (95:5, v/v) was selected for the extraction because this solvent mixture had high extraction efficiency, not only for coniferyl ferulate but also for ferulic acid, ligustilide and butylidenephthalide. In addition, 50-fold sample weight and 40 min were selected as the solvent volume and extraction time, respectively.

### Optimization of SFE

**Figure 2:**
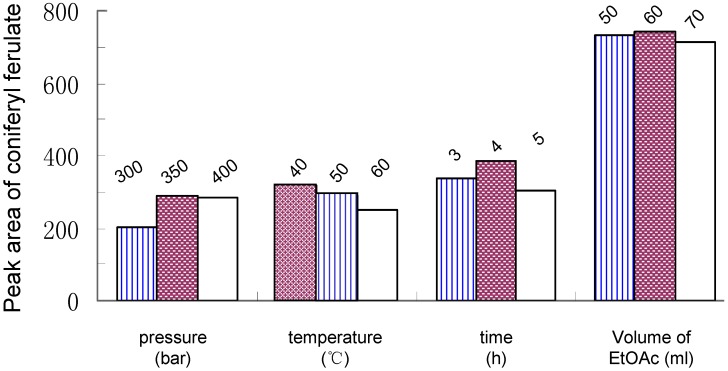
Effects of pressure, temperature, extraction time, volume of ethyl acetate on SFE of coniferyl ferulate from *Angelica sinensis*.

The parameters such as extraction pressure, temperature and time are usually the most important parameters for SFE. In a preliminary investigation, it was found that an organic modifier is necessary for improving the extraction efficiency of coniferyl ferulate from *A. sinensis*. Among the three solvents used (aqueous ethanol, ethanol and ethyl acetate), ethyl acetate was selected as the modifier because it had the highest extraction efficiency. Finally, the optimum conditions of SFE are as follows: pressure, 350 Bar; temperature, 40^o^C; static extraction time, 4 h, and volume of modifier, 60 mL (Figure 2).

### Identification and quantitation of coniferyl ferulate in extracts of A. sinensis

**Figure 3:**
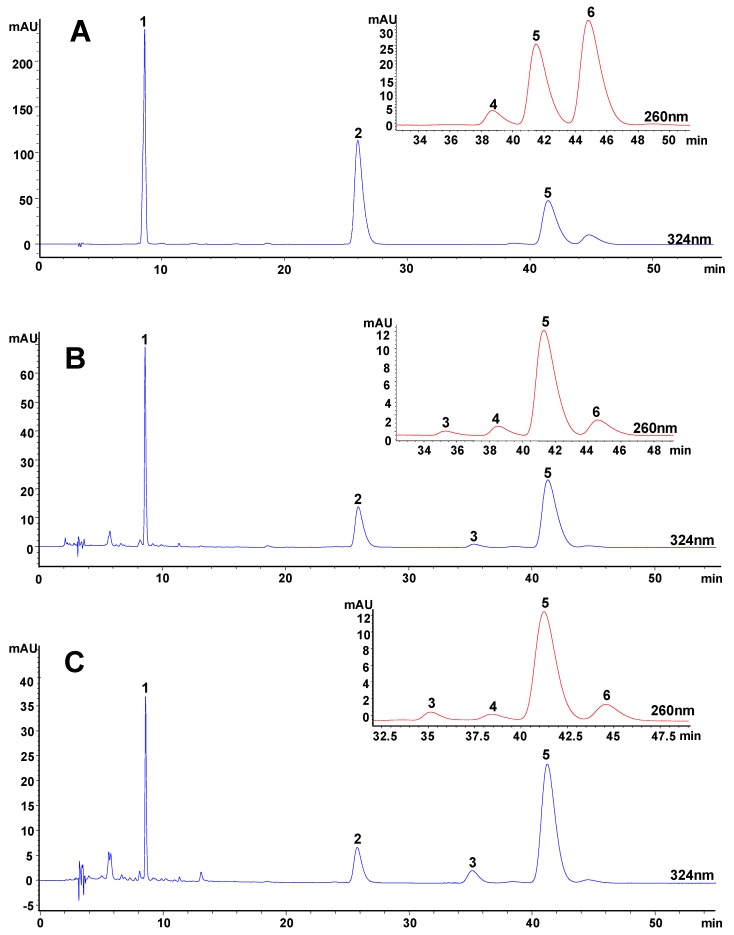

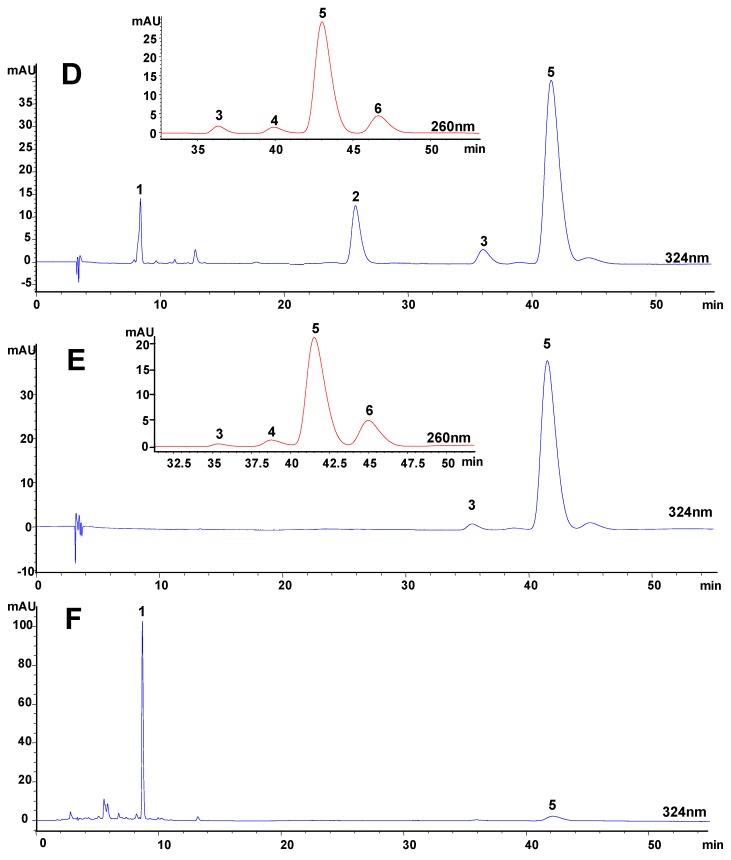
HPLC chromatograms of (A) mixed standards and typical extracts of *Angelica sinensis* extracted by (B) sonication extraction, (C) pressurized liquid extraction, (D) supercritical fluid extraction, (E) hydrodistillation and (F) decoction.

HPLC was used for quantitative analysis of coniferyl ferulate, ferulic acid, *E/Z*-ligustilide and *E/Z*-butylidenephthalide in the extracts of *Danggui* (Figure 3). The peaks of analytes were identified by two means: (i) by comparing the retention time of the peak with the reference compounds eluted under the same conditions and (ii) by spiking the sample with stock standard solution of analytes. In order to avoid the co-elution of analytes with interferents, the peak purities in chromatograms of extracts were determined by comparing their UV spectra with those of their standard compounds, which were also confirmed by their MS data.

**1**, ferulic acid; **2**, coniferyl ferulate; **3**, *E*-ligustilide; **4**, *E*-butylidenephthalide;

**5**, *Z*-ligustilide; **6**, *Z*-butylidenephthalide.

The contents of the analytes in the extracts were calculated by comparing to a known concentration of their standard solutions in the linear ranges, except the content of *E*-ligustilide was estimated using *Z*-ligustilide as the standard because of the absence of an *E*-ligustilide standard. The extractable contents of the six compounds in *Angelica sinensis* materials were shown in Table 1.

**Table 1:** Extractable contents (mg/g) of six compounds in *Angelica sinensis* using sonication (SE), pressurized liquid extraction (PLE), supercritical fluid extraction (SFE), hydrodistillation (HD) and decoction (DC).

Methods/Samples	Analytes (mean±SD, n=3)						
	FA ^a^	CF	*E*-Lig	*E*-Bp	*Z*-lig	*Z*-Bp	Total
**SE**							
OS ^b^	0.36±0.01	6.11±0.07	0.23±0.00	0.08±0.00	4.34±0.04	0.20±0.00	11.32
FS	0.10±0.00	20.12±0.47	0.84±0.02	0.10±0.00	11.85±0.23	0.10±0.00	33.11
**PLE**							
OS	0.44±0.01	6.18±0.19	0.51±0.01	0.11±0.01	6.96±0.13	0.22±0.01	14.42
FS	0.17±0.00	19.17±0.09	0.69±0.01	0.12±0.00	10.91±0.10	0.12±0.00	31.18
**SFE**							
OS	0.14±0.00	5.50±0.04	0.34±0.01	0.07±0.00	5.49±0.09	0.15±0.01	11.69
FS	0.05±0.00	8.65±0.06	0.32±0.01	0.05±0.00	5.63±0.13	0.04±0.00	14.74
**HD**							
OS	- ^c^	-	0.04±0.00	0.05±0.00	3.49±0.10	0.14±0.00	3.72
FS	-	-	0.04±0.00	0.02±0.00	2.60±0.05	0.04±0.00	2.70
**DC**							
OS	0.76±0.02	-	0.04±0.00	-	0.53±0.02	-	1.33
FS	1.04±0.04	-	0.08±0.00	-	0.55±0.01	-	1.67

^a^FA, ferulic acid; CF, coniferyl ferulate; *E*-Lig, *E*-ligustilide; *E*-Bp, *E*-butylidenephthalide; *Z*-Lig, *Z*-ligustilide; *Z*-Bp, *Z*-butylidenephthalide; ^b^OS: old stock sample; FS, fresh sample; refer to *Angelica sinensis* obtained from Minxian of Gansu Province in November of 2005 and 2006, respectively; ^c^ Not detected.

### Comparison of the five methods of extraction of coniferyl ferulate from A. sinensis

**Figure 4:**
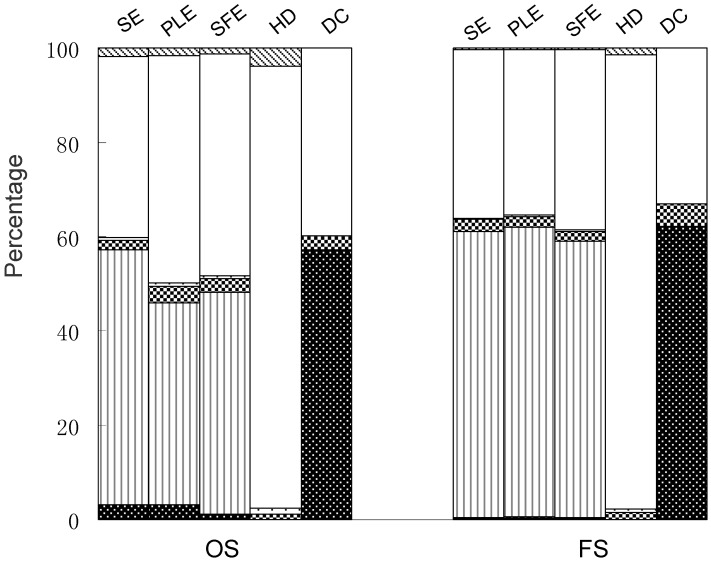
Comparison of six investigated compounds in different extracts from *Angelica sinensis* (old stock sample (OS) and fresh sample (FS)) extracted by sonication extraction (SE), pressurized liquid extraction (PLE), supercritical fluid extraction (SFE), hydrodistillation (HD) and decoction (DC).

It has been reported that coniferyl ferulate has multiple pharmacological activities [17,18,19,20]. Therefore, it is necessary and important for optimization of the extraction of coniferyl ferulate from medicinal plants such as *A. sinensis* because for its known instability. Our results show that the order of extraction efficiency is: SE≈PLE>SFE>>HD, DC. The composition of the SE, PLE and SFE extracts were very similar and all had high ratios of coniferyl ferulate. Coniferyl ferulate, however, was not obtained by HD and DC, though they had high selectivity for the extraction of ligustilide and ferulic acid, respectively (Figure 4). On the other hand, in Traditional Chinese Medicine practice, the usual administration form is *via* decoction, so it is noteworthy that the contents of ligustilide and coniferyl ferulate in the decoction were so low that they were not detected. This suggested that they might contribute little to the clinical efficacy of *A. sinensis*, though both compounds are reported to possess multiple pharmacological activities [12,13,17,18,19,20]. Especially, it is very interesting that the decoction extract only contains a little amount of ferulic acid as coniferyl ferulate, which in abundant in *A. sinensis* in and can be degraded into the former. Coniferyl ferulate could not even be detected in the decoction (data not shown). It was suggested that some other degraded compounds might be produced, which needs further investigation. Actually, it is thought that interaction of multiple chemical compounds contributes to the therapeutic effects of Chinese medicines. The overall clinical efficacy of these extracts has not been determined. Therefore, comparison of chemical components and pharmacological activities of these extracts is helpful to elucidate the mechanism of therapeutic effects and active components in *A. sinensis*.

()*Z*-butylidenephthalide, ()*Z*-Ligustilide, ()*E*-butylidenephthalide, ()*E*-ligustilide, ()coniferyl ferulate, ()ferulic acid.

In conclusion, five methods for extraction of coniferyl ferulate as well as ferulic acid, *E/Z*-ligustilide and *E/Z*-butylidenephthalide from *A. sinensis* were optimized and compared. Our results suggested that SE and PLE had much higher extraction efficiency, while HD and DC had good selectivity for the extraction of ligustilide and ferulic acid, respectively. Coniferyl ferulate was not obtained by HD and DC.

## Experimental

### Materials

The roots of *A. sinensis*, identified by the corresponding author, were obtained from Minxian of Gansu Province in November of 2005 (old stock sample, OS) and 2006 (fresh sample, FS), respectively. All the voucher specimens of *A. sinensis* root were deposited at the Institute of Chinese Medical Sciences, University of Macau, Macau SAR, P.R. China. Ferulic acid, *Z*-butylidenephthalide and *E*-butylidenephthalide were purchased from Sigma (St. Louis, MO, USA). *Z*-ligustilide was purchased from Chroma-Dex (Santa Ana, CA, USA). Coniferyl ferulate was separated and purified from essential oil of *A. sinensis* in our lab. Its structure was confirmed by comparison of its UV, MS, ^1^H-NMR and ^13^C-NMR data with the literature [22,23]. The purity tested by HPLC was more than 95%. Acetic acid and acetonitrile for liquid chromatography were purchased from Merck (Damstadt, Germany). Water was prepared using a Millipore Milli-Q Plus system (Millipore, Bedford, MA, USA). The reagents not mentioned here were from standard sources.

### Hydrodistillation

HD was performed according to the method described in the Chinese Pharmacopoeia [24]. Fifty gram of *A. sinensis* powder (0.32-0.43 mm) was placed in the flask of a Clevenger extractor and extracted with water (250 mL) for 6 h. The water suspension was extracted with ethyl acetate thrice (15 mL). The ethyl acetate extract and the essential oil obtained were pooled and transferred to a 50 mL volumetric flask and made up to its volume with acetonitrile. Then an aliquot of the solution (2 mL) was drawn into a 50 mL volumetric flask and made up to its volume with acetonitrile to obtain the sample solution. Finally, the solution was filtered through a 0.45 μm Econofilter (Agilent Technologies) before HPLC analysis.

### Sonication extraction

Sonication extraction (SE) was performed using an ultrasonic cleaning bath (Model 8510E – DTH, Branson, USA) as described by Li *et al.* with modification [25]. *A. sinensis* powder (0.5 g, 0.09-0.13 mm) was placed in a 50 mL flask, methanol-formic acid (25 mL, 95:5, v/v) was added and then the solution was weighed. SE (43 kHz, 320 W) was performed for 40 min at room temperature, then the mixture was made up to its original weight by addition of the same solvent, and then the supernatant was filtered through a 0.45 μm Econofilter prior to injection into the HPLC system.

### Decoction

Decoction (DC) is the traditional extraction method for oral administration of Chinese medicines. A solid–liquid extractor (Model KHS1, IKA-Werke, Staufen, Germany) was applied for the decoction which was performed according to the conventional method for clinical use. In brief, sliced crude drug of *A. sinensis* (5 g) was placed in the heating bottle and soaked in water (50 mL) for 30 min after weighing. Then the mixture was quickly heated to boiling and the temperature was kept at about 98-100 °C for 20 min. The extract was cooled to room temperature and water was added to make up the loss of weight. Centrifugation was performed at 4,000 rpm for 10 min and the supernatant was collected. The residue was extracted again with water (30 mL) for 15 min with the same method mentioned above. The two supernatants were pooled and filtered through a 0.45 μm Econofilter before HPLC analysis.

### Supercritical fluid extraction

A SFT-250 SFE/SFR system (Supercritical Fluid Technologies, Newark, DE, USA) was used for the extraction. The parameters, including pressure, temperature, static time and volume of ethyl acetate (modifier selected based on the preliminary investigation), which influence the extraction efficiency of SFE, were optimized using a univariate design approach. In brief, the volume of ethyl acetate was optimized after the other three parameters determined. During optimization of pressure, temperature and static time, the other two parameters were kept at the second level (pressure, 350 bar; temperature, 50 °C; static time, 4 h) when one parameter was optimized. The extraction was performed by filling the 100 mL extraction vessel with *A. sinensis* powder (20.0 g, 0.09-0.13 mm) under specific conditions, and the extracts were collected in a 50 mL volumetric flask and made up to its volume with acetonitrile. After appropriate dilution, the sample solution was filtered through a 0.45 μm Econofilter before HPLC analysis.

### Pressurized liquid extraction

Pressurized liquid extractions were performed on a Dionex ASE 200 (Dionex Corp., Sunnyvale, CA, USA) system as described by Lao *et al* with modification [26]. In brief, *A. sinensis* powder (0.3 g, 0.09-0.13 mm) was mixed with diatomaceous earth (2 g) and placed into an 11 mL stainless steel extraction cell. The use of a dispersion agent, such as diatomaceous earth, is recommended in order to reduce the solvent volume used for the extraction and prevent the aggregation of sample particles and the blockage of extraction cell outlet. The extraction cells were extracted under the following conditions: solvent, methanol; temperature, 100 °C; static extraction time, 10 min; pressure, 1,200 p.s.i.; static cycle, 2 and 60% of the flush volume. The extract was transferred to a 25 mL volumetric flask and made up to its volume with extraction solvent. The sample solution was filtered through a 0.45 μm Econofilter before HPLC analysis.

### HPLC analysis

Quantitative analysis of six compounds in the extracts of *A. sinensis* were performed on a Agilent Series 1100 liquid chromatograph, equipped with a vacuum degasser, a quaternary pump, an autosampler and a DAD detector, connected to a Agilent ChemStation software [27]. In brief, a ZORBAX ODS C_18_ column (4.6 mm × 250 mm i.d., 5 μm) and a ZORBAX ODS C_18_ guard column (4.6 mm×12.5 mm i.d., 5 μm) were used. Solvents that constituted the mobile phase were A (1% aqueous acetic acid) and B (acetonitrile). The elution conditions applied were: 0-7 min, linear gradient 10-33% B; and 7-55 min, isocratic elution of 33% B. Flow rate was 1.0 ml/min and the injection volume was 10 μl. The system operated at 25 °C. Peaks were detected at 324 nm for ferulic acid, coniferyl ferulate, *E/Z*-ligustilide and 260 nm for *E/Z*-butylidenephthalide. The method was validated based on the standard curves, limit of detection, limit of quantitation, precision, stability, repeatability and recovery of the six investigated compounds, which suggested that method was available for quantitative determination of the analytes [27].

## References

[1] Liu L.N., Mei Q.B., Cheng J.F., Li X. (2002). Study on anti-dysmenorrhea effect of essential oil from *Angelica sinensis* Diels. Jie Fang Jun Yao Xue Xue Bao.

[2] Mei Q.B., Tao J.Y., Cui B. (1991). Advances in the pharmacological studies of *Angelica sinensis* (Oliv) Diels (Chinese Danggui). Chin. Med. J..

[3] National Committee of Chinese Pharmacopoeia (2005). Pharmacopoeia of the People’s Republic of China.

[4] Hu H.J., Hang B.Q., Wang P.S. (1991). Anti-inflammatory effect of Radix *Angelicae Sinensis*. Zhong Guo Zhong Yao Za Zhi.

[5] Wu S.J., Ng L.T., Lin C.C. (2004). Antioxidant activities of some common ingredients of traditional Chinese medicine, *Angelica sinensis*, *Lycium barbarum* and *Poria coco*. Phytother. Res..

[6] Yang T.H., Jia M., Meng J., Wu H., Mei Q.B. (2006). Immunomodulatory activity of polysaccharide isolated from *Angelica Sinensis*. Int. J. Biol. Macromol..

[7] Huang W.H., Song Q.C. (2001). The progress of chemical and pharmacological research on *Angelica sinensis*. Zhong Guo Zhong Yao Za Zhi.

[8] Cao W., Li X.Q., Liu L., Yang T.H., Li C., Fan H.T., Jia M., Lv Z.G., Mei Q.B. (2006). Structure of an anti-tumor polysaccharide from *Angelica sinensis* (Oliv.) Diels. Carbohydr. Polym..

[9] Xia Q., Zhang P., Li S.P., Wang Y. T. (2004). The research on pharmacological effects of *Angelica sinensis*. Shi Zhen Guo Yi Guo Yao.

[10] Lin L.Z., He X.G., Lian L.Z., King W., Elliott J. (1998). Liquid chromatographic– electrospray mass spectrometric study of the phthalides of *Angelica sinensis* and chemical changes of Z-ligustilide. J. Chromatogr. A.

[11] Ou S.Y., Bao H.Y., Lan Z.D. (2001). The pharmacological research progress of ferulic acid and its derivatives. Zhong Yao Cai.

[12] Du J.R., Yu Y., Yao Y., Bai B., Zong X., Lei Y., Wang C.Y., Qian Z.M. (2007). Ligustilide reduces phenylephrine induced-aortic tension in vitro but has no effect on systolic pressure in spontaneously hypertensive rats. Am. J. Chin. Med..

[13] Tao J.Y., Ruan Y.P., Mei Q.B., Liu S., Tian Q.L., Chen Y.Z., Zhang H.D., Duan Z.X. (1984). Studies on the antiasthmatic action of ligustilide of Dang-gui, *Angelica sinensis* (oliv.) diels. Yao Xue Xue Bao.

[14] Teng C.M., Chen W.Y., Ko W.C., Ouyang C. (1987). Antiplatelet effect of butylidenephthalide. Biochim. Biophys. Acta.

[15] Ou S.Y. (2002). The function and application of ferulic acid. Xian Dai Shi Pin Ke Ji.

[16] Lu G.H., Chan K., Leung K., Chan C.L., Zhao Z.Z., Jiang Z.H. (2005). Assay of free ferulic acid and total ferulic acid for quality assessment of *Angelica sinensis*. J. Chromatogr. A.

[17] Zou H.F., Kong L., Sun N.H., Wu L.H., Chen X.G. (2005). Use of coniferyl ferulate in anticancer medicine. China Patent.

[18] Chou S.C., Everngam M.C., Sturtz G., Beck J.J. (2006). Antibacterial activity of components from *Lomatium californicum*. Phytother. Res..

[19] Naito T., Iketani Y., Kubota K., Shimoda Y. (1995). Vasodilators containing coniferyl ferulate and phthalide dimers of *Cnidium officinale*. Jap. Pat..

[20] Li S.Y., Yu Y., Li S.P. (2007). Identification of antioxidants in essential oil of Radix *Angelicae sinensis* using HPLC coupled with DAD-MS and ABTS-based assay. J. Agric. Food. Chem..

[21] Kong L., Yu Z.Y., Bao Y.M., Su X.Y., Zou H.F., Li X. (2006). Screening and analysis of an antineoplastic compound in *Rhizoma Chuanxiong* by means of in vitro metabolism and HPLC-MS. Anal. Bioanal. Chem..

[22] Yang F., Xiao Y.S., Zhang F.F., Xue X.Y., Xu Q., Liang X.M. (2006). High performance liquid chromatography-mass spectrometry analysis of Radix *Angelica sinensis*. Yao Xue Xue Bao.

[23] Lu X.H., Zhang J.J., Liang H., Zhao Y.Y. (2004). Chemical Constituents of *Angelica sinensis*. Chin. J. Pharm. Sci..

[24] National Committee of Chinese Pharmacopoeia (2005). Pharmacopoeia of the People’s Republic of China.

[25] Li P., Li S.P., Lao S.C., Fu C.M., Kan K.K.W., Wang Y.T. (2006). Optimization of pressurized liquid extraction for Z-ligustilide, Z-butylidenephthalide and ferulic acid in *Angelica sinensis*. J. Pharm. Biomed. Anal..

[26] Lao S.C., Li S.P., Kan K.K.W., Li P., Wan J.B., Wang Y.T., Dong T.T.X., Tsim K.W.K. (2004). Identification and quantification of 13 components in *Angelica sinensis* (Danggui) by gas chromatography–mass spectrometry coupled with pressurized liquid extraction. Anal. Chim. Acta.

[27] Xie J.J., Yu Y., Wang Y.T., Li S.P. (2007). Simultaneous HPLC determination of 6 components in *Angelica sinensis*. Yao Wu Fen Xi Za Zhi.

